# ICG fluorescence imaging in colorectal surgery: a snapshot from the ICRAL study group

**DOI:** 10.1186/s12893-021-01191-6

**Published:** 2021-04-10

**Authors:** Gian Luca Baiocchi, Gianluca Guercioni, Nereo Vettoretto, Stefano Scabini, Paolo Millo, Andrea Muratore, Marco Clementi, Giuseppe Sica, Paolo Delrio, Graziano Longo, Gabriele Anania, Vittoria Barbieri, Pietro Amodio, Carlo Di Marco, Gianandrea Baldazzi, Gianluca Garulli, Alberto Patriti, Felice Pirozzi, Raffaele De Luca, Stefano Mancini, Corrado Pedrazzani, Matteo Scaramuzzi, Marco Scatizzi, Lucio Taglietti, Michele Motter, Graziano Ceccarelli, Mauro Totis, Andrea Gennai, Diletta Frazzini, Gianluca Di Mauro, Gabriella Teresa Capolupo, Francesco Crafa, Pierluigi Marini, Giacomo Ruffo, Roberto Persiani, Felice Borghi, Nicolò de Manzini, Marco Catarci

**Affiliations:** 1 Department of Clinical and Experimental Sciences, University of Brescia, ASST Spedali Civili, Brescia, Italy; 2 General Surgery Unit, CG Mazzoni Hospital, Ascoli Piceno, Italy; 3 General Surgery Unit, ASST Spedali Civili, Montichiari, BS Italy; 4 General & Oncologic Surgery Unit, National Cancer Center “San Martino”, Genova, Italy; 5 General Surgery Unit, Aosta Regional Hospital, Aosta, Italy; 6 General Surgery Unit, Agnelli Hospital, Pinerolo, TO Italy; 7 General Surgery Unit, University Hospital, L’Aquila, Italy; 8 General Surgery Unit, Policlinico Tor Vergata University Hospital, Roma, Italy; 9 Colorectal Surgical Oncology Unit, IRCCS G. Pascale Foundation, Napoli, Italy; 10 General Surgery Unit, Policlinico Casilino, Roma, Italy; 11 General Surgery Unit, University Hospital, Ferrara, Italy; 12 General Surgery Unit, Cardinale G. Panico Hospital, Tricase, LE Italy; 13 General Surgery Unit, Belcolle Hospital, Viterbo, Italy; 14 General Surgery Unit, Conegliano Hospital (TV) ULSS2 Marca Trevigiana, Conegliano, Italy; 15 General Surgery Unit, ASST Nord Hospital, Sesto San Giovanni, MI Italy; 16 General Surgery Unit, Infermi Hospital, Rimini, Italy; 17 General Surgery Unit, Marche Nord Hospital, Pesaro e Fano, PU Italy; 18 General Surgery Unit, ASL Napoli2 Hospital, Pozzuoli, NA Italy; 19 General Surgery Unit, IRCCS Istituto Giovanni Paolo II, Bari, Italy; 20 General & Oncologic Surgery Unit, San Filippo Neri Hospital, Roma, Italy; 21 General Surgery Unit, University Hospital, Verona, Italy; 22 General Surgery Unit, IRCCS Casa Sollievo Della Sofferenza, San Giovanni Rotondo, FG Italy; 23 General Surgery Unit, Santa Maria Annunziata Hospital, Firenze, Italy; 24 General Surgery Unit, ASST Valle Camonica, Esine, Italy; 25 General Surgery Unit 1, Santa Chiara Hospital, Trento, Italy; 26 General Surgery Unit, San Giovanni Battista Hospital, Foligno, PG Italy; 27 General Surgery Unit, San Gerardo Hospital, Monza, Italy; 28 General Surgery Unit, Sant’Andrea Hospital, La Spezia, Italy; 29 General Surgery Unit, Ospedale Civile Di Pescara, Pescara, Italy; 30 General Surgery Unit, Ospedale Di Ragusa, Ragusa, Italy; 31 Colorectal Surgery Unit, Policlinico Universitario Campus Bio Medico, Roma, Italy; 32 General & Oncologic Surgery Unit, San Giuseppe Moscati Hospital, Avellino, Italy; 33 General Surgery Unit, San Camillo Hospital, Roma, Italy; 34 General Surgery Unit, IRCCS Sacro Cuore Don Calabria Hospital, Negrar di Valpolicella, VR Italy; 35 Minimally Invasive Oncologic Surgery Unit, IRCCS Policlinico Gemelli Foundation, Roma, Italy; 36 General Surgery Unit, Santa Croce E Carle Hospital, Cuneo, Italy; 37 General Surgery Unit, University Hospital, Trieste, Italy

**Keywords:** Colon cancer, Rectal cancer, Laparoscopy, Fluorescence guided surgery, ICG

## Abstract

**Background:**

Fluorescence-guided visualization is a recently proposed technology in colorectal surgery. Possible uses include evaluating perfusion, navigating lymph nodes and searching for hepatic metastases and peritoneal spread. Despite the absence of high-level evidence, this technique has gained considerable popularity among colorectal surgeons due to its significant reliability, safety, ease of use and relatively low cost. However, the actual use of this technique in daily clinical practice has not been reported to date.

**Methods:**

This survey was conducted on April 2020 among 44 centers dealing with colorectal diseases and participating in the Italian ColoRectal Anastomotic Leakage (iCral) study group. Surgeons were approximately equally divided based on geographical criteria from multiple Italian regions, with a large proportion based in public (89.1%) and nonacademic (75.7%) centers. They were invited to answer an online survey to snapshot their current behaviors regarding the use of fluorescence-guided visualization in colorectal surgery. Questions regarding technological availability, indications and techniques, personal approaches and feelings were collected in a 23-item questionnaire.

**Results:**

Questionnaire replies were received from 37 institutions and partially answered by 8, as this latter group of centers do not implement fluorescence technology (21.6%). Out of the remaining 29 centers (78,4%), fluorescence is utilized in all laparoscopic colorectal resections by 72.4% of surgeons and only for selected cases by the remaining 27.6%, while 62.1% of respondents do not use fluorescence in open surgery (unless the perfusion is macroscopically uncertain with the naked eye, in which case 41.4% of them do). The survey also suggests that there is no agreement on dilution, dosing and timing, as many different practices are adopted based on personal judgment. Only approximately half of the surgeons reported a reduced leak rate with fluorescence perfusion assessment, but 65.5% of them strongly believe that this technique will become a minimum requirement for colorectal surgery in the future.

**Conclusion:**

The survey confirms that fluorescence is becoming a widely used technique in colorectal surgery. However, both the indications and methods still vary considerably; furthermore, the surgeons' perceptions of the results are insufficient to consider this technology essential. This survey emphasizes the need for further research to reach recommendations based on solid scientific evidence.

## Background

Indocyanine green (ICG), approved for clinical use by the Food and Drug Administration (FDA) since 1959, is the most commonly used fluorescent probe. It is a low-cost molecule that is easy to use, widely available and negligibly toxic [1]. ICG binds primarily to serum albumin and other serum globulins, such as alpha-1 lipoprotein, and then circulates, behaving like a macromolecule [2]. In recent years, ICG fluorescence-guided visualization has gained a predominant role in visceral surgery. With particular reference to colorectal surgery, the main use of this technology is in real-time intraoperative angiography, which allows us to assess the perfusion of anastomotic stumps before and after anastomosis [3]. Another possible use is the search for superficial liver metastases [4] and small peritoneal metastases after intravenous injection performed at different times before surgery [5]; finally, intratumor injection of ICG allows visualization of the tumor itself and the draining of regional lymph nodes [6]. The wide availability of this technology (now present in nearly all new laparoscopic systems), its ease of use, the low cost of the molecule and the excellent visual yield have caused this technology to spread rapidly, despite the lack of robust scientific evidence from randomized controlled clinical trials.

The Italian ColoRectal Anastomotic Leakage (iCral) Study Group comprises Italian surgeons particularly interested and experienced in colorectal surgery [7–9] working in 44 centers from nearly all Italian regions. It represents, therefore, an ideal background for a survey aimed at showing a snapshot of the current diffusion of ICG fluorescence-guided technology in Italy.

The aim of this study is to obtain a picture of how and to what extent ICG fluorescence-guided visualization is used in colorectal surgery to understand if the indications and the methods of use are sufficiently consistent and uniform to make prospective clinical studies unnecessary or outdated by daily clinical practice. The secondary aims are to analyze the availability of the technology, to detect organizational problems and highlight unsolved problems and to collect colorectal surgeons’ opinions about the actual usefulness and future developments of this technology.

## Methods

A questionnaire including 23 items was sent to 44 iCral study group centers. The study protocol was performed in accordance with the relevant guidelines. The Institutional Review Board of the University of Brescia, Department of Clinical and Experimental Sciences, approved the study protocol. A waiver of written informed consent was granted by the ethics committee of the University of Brescia.

The questionnaire was structured in 4 sections: (1) hospital features (number of beds, level of care, mission), and surgical unit size (number of operating rooms and intensive care unit (ICU) hallmarks); (2) availability of fluorescence technology (brand, number of laparoscopic columns, effective availability in terms of number specialties sharing columns and contemporary multiple operations with fluorescence); (3) indications for use, related to type of intervention, type of surgical approach, type of patient; (4) dosage, dilution, injection timing; and (5) personal opinion about usefulness of fluorescence technology and future developments. Maximum time granted to answer was 2 days.

The datasets used and analyzed during the current study are available from the corresponding author on request.

## Results

Questionnaire replies were received from 37 institutions (response rate 84.1%). Table 1 reports the features of the participating institutions. Surgeons were approximately equally divided on the basis of geographical criteria, with a large proportion working out of public (89.1%) and nonacademic (75.7%) centers. More than 50 cases/year were declared by 89,2% of centers (70.3% declared more than 80 cases/year). Only 21.6% of the centers have no availability for fluorescence technology; therefore, the analysis was conducted on the remaining 78.4% of centers (10.8% of them had the technology available only on a trial basis). Table 2 describes the availability and technical characteristics of the utilized tools. More than half of the respondents always have the technology available, including for contemporary or same-day surgery (62.1% of centers), and have the ICG vials in the OR (96.5%). Seven centers have more than one laparoscopic system that is capable of visualizing fluorescence. Table 3 provides data on indications for fluorescence utilization. Only 27.6% of surgeons said that the use of fluorescence is limited to selected cases, while the remaining surgeons use the fluorescence to assess perfusion in all interventions. However, this is limited to the laparoscopic approach: only 37.9% of surgeons also utilize fluorescence in open interventions (usually by laparoscopic instruments). The few surgeons who described a selective utilization of fluorescence usually program the utilization before starting the intervention for high-risk patients (37,5%) and/or for high-risk interventions (43,7%); only 18.7% of them pick up the instruments during the intervention, in cases of uncertain perfusion. Approximately half of the survey participants use fluorescence only for perfusion evaluation, while 35.5% also use it to mark the site of the neoplasm, and 16.1% identify potentially affected lymph nodes. None of the respondents uses fluorescence to search for small peritoneal carcinoses that cannot be seen with the naked eye. The majority (78.6%) of those who also use fluorescence for purposes other than perfusion evaluation do not believe that it limits the quality of perfusion evaluation.

**Table 1:** The thirty-seven institutions that answered the survey

	Survey answers	Num	%
Type of hospital	Public, academic	9	24.3
	Public, nonacademic	24	64.8
	Private	4	10.9
Region	Northern Italy	21	56.7
	South-central Italy	16	43.2
Total number of beds	< 201	2	5.4
	201–500	18	48.6
	501–1000	11	29.7
	> 1000	6	16.3
Number of ORs/week	< 3	4	10.8
	4–5	7	18.9
	6–10	18	48.6
	> 11	8	21.6
Colorectal cancer cases/year	< 50	4	10.8
	50–80	7	18.9
	> 80	26	70.3
Technology for fluorescence available in 2019	No	8	21.6
	Yes, on trial	4	10.8
	Yes	25	67.6

**Table 2:** Availability of fluorescence technology in 29 colorectal surgical units

		Num	%
Fluorescence system (7 centers have more than 1 system)^1^	Karl Storz	13	44.8
	Stryker	5	17.2
	Surgical Intuitive/Firefly	8	27.6
	Olympus	9	31.0
	Novadaq	3	10.3
Number of fluorescence systems in multidisciplinary ORs	1	9	31.0
	2	16	55.2
	> 2	4	13.8
Fluorescence system always available for general surgery ORs	Yes	17	58.6
	No	12	41.4
Fluorescence system available for more than 1 simultaneous colorectal intervention	Yes	18	62.1
	No	11	37.9
Is ICG (Verdye, 25 mg) always available in the OR?	Yes	28	96.5
	No	1	3.5

^1^More than one answer accepted

**Table 3:** Clinical indications for fluorescence perfusion assessment

		Num	%
What colorectal operation is scheduled with fluorescence technology?	All colorectal resections	14	48.3
	All colorectal resections, if available	7	24.1
	In selected cases	8	27.6
Selected cases in which fluorescence perfusion is utilized^1^	High risk patient	6	75.0
	High risk intervention (rectum/transverse)	7	87.5
	Intraoperative doubtful perfusion	3	37.5
Does the surgical approach matter? Fluorescence is utilized:	In both open surgery and laparoscopy	11	37.9
	Only in laparoscopy	6	20.7
	In laparoscopy and in open uncertain cases	12	41.4
Fluorescence perfusion assessment is most important in:^2^	Rectal resection	8	27.6
	Transverse colon and left flexure resection	11	37.9
	Extended right or left hemicolectomy	10	34.5
Is fluorescence used for other purposes in colorectal resection? ^3^	Yes, nodal navigation	5	17.2
	Yes, peritoneal carcinomatosis assessment	0	0
	Yes, tumor tattooing	11	37.9
	No	15	51.7
In case of tumor marking by ICG, is perfusion assessment impaired?	Yes	3	21.4
	No	11	78.6

^1^Answers were provided only by 8 surgeons answering “In selected cases” to the previous question. More than one answer was accepted

^2^Only one answer was accepted

^3^More than one answer was accepted

From a technical perspective (Table 4), there was no agreement on how to use fluorescence for perfusion evaluation. The dilutions vary from 0.25 to 2.5 mg/ml (51.8% use the lower dilution), and the dose is independent of weight for 79.3% of surgeons but varies from 5 mg (most commonly used dose, 39.1% of surgeons) to 25 mg. The remaining 20.7% of respondents use weight-based dosing: the majority of these respondents use 0.2–0.3 mg/kg. Finally, even on the timing of the injection (1, 2 or 3 injections,before or after the anastomosis,laparoscopically or endoscopically), there was no clear preference.

**Table 4:** Technical characteristics of perfusion assessment by fluorescence

		Num	%
Dilution	0.25 mg/ml	4	13.8
	0.5 mg/ml	5	17.2
	1 mg/ml	5	17.2
	2,5 mg/ml	15	51.8
ICG dosing	Standard	23	79.3
	Dependent on patient weight	6	20.7
Standard ICG dose	5 mg	9	39.2
	10 mg	5	21.7
	15 mg	3	13.1
	20 mg	1	4.3
	25 mg	5	21.7
Patient weight-dependent ICG doses	0.1 mg/kg	1	16.7
	0.2–0.3 mg/kg	4	66.6
	> 0.3 mg/kg	1	16.7
Timing of injection in ileocolic and colocolic anastomosis	Before proximal and distal colon Sect. (1 injection)	14	48.3
	Before proximal and distal colon section/after anastomosis (2 injections)	13	44.8
	After anastomosis (1 injection)	2	6.9
Timing of injection in colorectal anastomosis	Before proximal colon Sect. (1 injection)	8	27.6
	Before proximal colon section/before anastomosis (2 injections)	13	44.9
	Before proximal colon section/after anastomosis by endoscopy (2 injections)	3	10.3
	Before proximal colon section/before anastomosis/after anastomosis by endoscopy (3 injections)	5	17.2

Participating surgeons were also asked for their opinion on the effectiveness of this method (Table 5). Only 41.4 and 55.2% stated that visceral perfusion assessment reduced the leak rate (statistically and based on personal nonstatistical assessment, respectively). However, 65.5% of them believe that in the future, this method will become mandatory, especially in the face of medico-legal considerations. Finally, there is no agreement on which type of intervention benefits most from the technology (Table 3).

**Table 5:** General opinion on the value of fluorescence in colorectal surgery

		Num	%
In your experience, does perfusion assessment by fluorescence reduce anastomotic leak rate by statistical analysis?	Yes	12	41.4
	No	17	58.6
In your experience, does perfusion assessment by fluorescence reduce anastomotic leak rate subjectively?	Yes	16	55.2
	No	13	44.8
Do you believe perfusion assessment by fluorescence will become a minimum requirement in colorectal surgery?	Yes	19	65.5
	No	10	34.5

## Discussion

Fluorescence-guided surgery allows enhanced real-time intraoperative visualization of anatomical structures and/or vascular perfusion [3–6]. In this technique, the operative field is exposed to near-infrared light after the target has been injected by indocyanine green, the most commonly used fluorescent dye. Fluorescence can be visualized both directly on the operative field and in open surgical procedures, both on the screen, and in minimally invasive procedures. Due to its low cost, ease of use and wide availability, in the last few years, fluorescence-guided surgery has been used in many surgical specialties [10, 11]. In colorectal procedures, one of the most feared complications is anastomotic leak, whose incidence is accepted at a 5–7% rate, even in high volume centers [12]; approximately half of anastomotic leaks are believed to be related to an insufficient vascular supply that is not detected with the naked eye while performing anastomosis. Bowel perfusion can be evaluated intraoperatively by an ICG intravenous injection and fluorescence detection. This ICG-based angiography might reveal the optimal resection site on both sides of the anastomosis (changes in the transection line are reported in 5–15% of cases [13]) and represents a promising technique for reducing the leak rate. Some phase II trials confirmed the feasibility, low cost and high success rate of this procedure [14–17], reporting a leakage rate (< 3%) lower than expected based on a historical series, with particular reference to rectal anastomosis. Randomized prospective controlled trials (RCTs), however, were either prematurely stopped [18] or failed to finally demonstrate a statistically significant reduction in anastomotic leaks [19]. Other RCTs will be published [20, 21], and some other efforts will be finalized to better understand the dynamic information on perfusion gleaned with this technology [22].

The main goal of this paper is to describe protocols for handling ICG fluorescence imaging in colorectal interventions within Italian Departments of General Surgery participating in the iCRAL group. Fluorescence-guided surgery has forcefully entered daily clinical practice without being supported by high-level scientific evidence that certifies its usefulness [23]. The implementation of a new technique without guidelines deriving from robust evidence can generate unjustified increases in health care costs and harm to patients. The road to achieving robust evidence passes through the design of prospective clinical trials, but these in turn must start from knowledge on current practices, which are significantly different from center to center. The lack of agreement on usage protocols makes it impossible to compare multicentric series. Before proposing prospective studies based on the rigid use of protocols, it is necessary to better understand current daily practices.

The present questionnaire reliably represents a cross-sectional snapshot of Italian centers performing colorectal surgery. The composition of the iCRAL Group reflects the national public/private hospital and the academic/nonacademic hospital relationship. Most of the involved surgeons work in medium to large hospitals (only 2 surgeons work in hospitals with fewer than 200 beds, and only 6 work in hospitals with more than 1000 beds). Participating institutions should be considered colorectal medium–high volume centers, with scientific and cultural interest on the subject [7–9]. Participation in the iCral study group requires that from 2017 onwards, all cases are included in a web-based prospective database, so the few questions in this survey that require a numerical analysis can be answered with immediately available and reliable data. The high rate of response to this questionnaire should be emphasized, as it is a testament to the great relevance of this issue among colorectal surgeons as well as to the cohesive spirit of this group.

The first consideration that emerges from the questionnaire analysis is that fluorescence technology is now extremely widespread: in 2019, only 21.6% of surgeons declared that they did not have the technology for fluorescence-guided surgery. Where it was available, in 69% of cases, there is more than one laparoscopic system; in 58.6% of cases, at least one column is always available; and in 62.1% of cases, multiple operations are possible at the same time. On the other hand, there is no consensus on the system brand; apart from a slight prevalence of Karl Storz devices, Stryker/Novadaq, Surgical Intuitive and Olympus units are equally distributed. We can therefore report a satisfactory diffusion of fluorescence technology, which makes it even more important to clarify the indications and techniques of use.

Regarding the indications, 72.3% of surgeons use fluorescence by default in all laparoscopic interventions, while only 37.9% use it by default in open procedures. On the other hand, 27.6% of surgeons consider fluorescence only in selected cases, but there is no agreement on which these are (high-risk patients, high-risk interventions, intraoperative uncertainty). Few surgeons also take into account features different from perfusion evaluation, such as lymph node navigation (16.1%) and tumor labeling (35.5%). Basically, the most widespread practice involves the use of fluorescence for anastomotic perfusion evaluation in all laparoscopic colorectal surgeries.

However, there is no agreement on the ideal timing for the perfusion assessment: for ileocolic anastomosis, approximately half of surgeons proceed with a single injection before the anastomosis, and half proceed with two injections one before and the second after the anastomosis (48.3% and 44.8%, respectively). For colorectal anastomosis, the majority of surgeons (44.9%) perform the injection before the proximal colon section and before the anastomosis, while 27.6% perform it only before the proximal colon section; 10.3% perform 2 injections, one of which is made endoscopically after the anastomosis, and 17.2% perform 3 injections. Further discrepancies are also found in dilution and dose, as reported in Table 4. It is evident that the discrepancy between these techniques limits the possibility of comparing the results between the different centers.

A final interesting note relates to the perception of Italian surgeons of the efficacy of fluorescence in preventing anastomotic leaks: surprisingly, only 65.5% believe that this method should be implemented to the point of becoming a minimum essential requirement in colorectal surgery. However, this is understandable if we consider the subjective perception and statistical evidence relating to the reduction in anastomotic leaks: as many as 44.8% or 58.6% of the interviewed surgeons declared that they did not detect a subjective or statistical reduction in leaks, respectively.

In conclusion, the assessment of visceral perfusion by means of fluorescence after an intravenous injection of ICG is an extremely widespread intraoperative technique yet is applied with considerable variability in protocols that prevents correct multicentric data collection aimed at a statistical demonstration of the usefulness of this procedure. Multilateral scientific action is required, coordinated and supported by the scientific societies of the surgical field to harmonize the application of the technique.

## Data Availability

The datasets used and/or analyzed during the current study are available from the corresponding author upon reasonable request.
